# Reviewer Acknowledgement 2012

**DOI:** 10.1186/1749-7922-8-9

**Published:** 2013-03-01

**Authors:** Fausto Catena, Frederick A Moore

**Affiliations:** 1 St Orsola- Malpighi University Hospital, Bologna, Italy; 2 University of Florida, Florida, United States of America

## Abstract

**Contributing reviewers:**

The Editors of *World Journal of Emergency Surgery* would like to thank all our reviewers who have contributed to the journal in Volume 7 (2012).

## 

Ferdinando Agresta

Italy

Ali Aminian

Iran

Darius Deo Balumuka

Tanzania

Sandro Barni

Italy

Jasneet Bhullar

United States of America

Walter Biffl

United States of America

Saptarshi Biswas

United States of America

L.D. Britt

United States of America

Desiree Burger

Netherlands

Clay Cothren Burlew

United States of America

Jill Cherry-Bukowiec

United States of America

Raul Coimbra

United States of America

Salomone Di Saverio

Italy

Samer Doughan

United Kingdom

Alex Escalona

Chile

Aristomenis K Exadaktylos

Switzerland

Alessandro Fancellu

Italy

Tatsuma Fukuda

Japan

Ralf Herbert Gahr

Germany

Athanasios Giannoukas

Greece

Sanjay Gupta

India

John Holcomb

United States of America

Rao Ivatury

United States of America

Nobuyasu Kano

Japan

Dimos Karangelis

United Kingdom

Kenji Kawamukai

Italy

Michael Kelly

Australia

Fernando Kim

United States of America

Yoram Kluger

Israel

Janusz Kowalewski

Poland

Rifat Latifi

United States of America

Philipp Lenzlinger

Switzerland

Celestino Pio Lombardi

Italy

Sheikh Muzamil

India

Takashi Nagata

Japan

Mehdi Ouaissi

France

Giorgio Rossi

Italy

Sandeep Sainathan

United States of America

Boris Sakakushev

Bulgaria

Özge Senyaman

Germany

R. Stephen Smith

United States of America

Korhan Taviloglu

Turkey

Tomislav Trupkovic

Germany

Gregorio Tugnoli

Italy

George Velmahos

United States of America

Suemoy Wallace

United States of America

Imtiaz Wani

India

